# Exploring the Effects of the Guanidinium:Methylammonium Ratio on the Photophysical Dynamics of ⟨*n*⟩ = 5 ACI Perovskites

**DOI:** 10.1021/acs.jpcc.5c04999

**Published:** 2025-10-01

**Authors:** Lisanne M. Einhaus, Xiao Zhang, Jeroen P. Korterik, Robert Molenaar, Guido Mul, Johan E. ten Elshof, Annemarie Huijser

**Affiliations:** † PhotoCatalytic Synthesis Group, MESA+ Institute for Nanotechnology, 3230 University of Twente, 7500 AE Enschede , The Netherlands; ‡ Inorganic Materials Science group, MESA+ Institute for Nanotechnology, 3230 University of Twente, 7500 AE Enschede, The Netherlands; § Optical Sciences Group, MESA+ Institute for Nanotechnology, 3230 University of Twente, 7500 AE Enschede, The Netherlands; ∥ NanoBioPhysics Group, MESA+ Institute for Nanotechnology, 3230 University of Twente, 7500 AE Enschede, The Netherlands

## Abstract

Quasi-two-dimensional (quasi-2D) lead halide perovskites with alternating cations in the interlayer (ACI) space represent a promising type of material for optoelectronics. Similar to the Ruddlesden–Popper and Dion–Jacobson types of perovskites, domains with different thicknesses (*n*) and bandgaps are formed within a single film. This work focuses on ⟨*n*⟩ = 5 ACI perovskites based on guanidinium (GA^+^) and methylammonium (MA^+^) cations and investigates the influence of the GA:MA ratio in the interlayer space on the photophysical processes after photoexcitation. Using a combination of time-resolved photoluminescence (TRPL) and femtosecond transient absorption (TA) spectroscopy, hot carrier cooling, the occurrence and directionality of energy or charge transfer between the different domains, and the exciton and charge carrier dynamics are studied and modeled using target analysis. After the thermalization of hot carriers and excitons, exciton transfer from low-*n* to high-low-*n* domains occurs within 10 ps, after which they dissociate into free charges. From there, charge transfer into the intermediate-*n* domains occurs in about 22–54 ps. In the layers with excess GA, this process possibly occurs in an undesirable competition with self-trapped exciton (STE) formation. From the intermediate-*n* domains, charges are transferred into the high-*n* domains in 95–159 ps, which process occurs the fastest in the GA-MA layer. Finally, charge carriers decay intrinsically on the nanosecond scale with the longest lifetimes for the GA-MA and GA-2MA systems, which is beneficial for PV applications.

## Graphical abstract

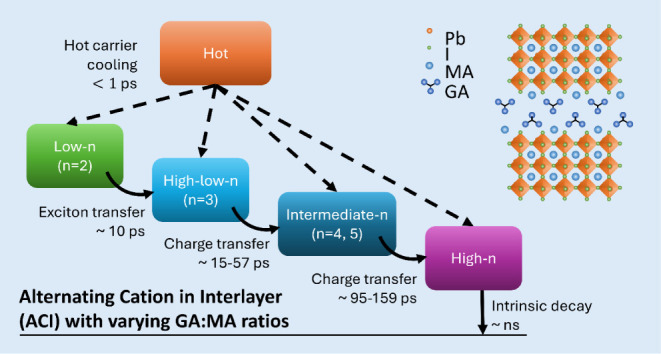

## Introduction

Metal halide perovskite has become a promising candidate to be used as an active material in optoelectronic devices, such as light-emitting diodes and photovoltaics (PV). Recently, quasi-two-dimensional (quasi-2D) lead iodide perovskites have gained significant attention due to their enhanced thermodynamic and environmental stability compared to the extensively studied three-dimensional (3D) perovskites.^1–7^ In quasi-2D perovskites, large organic spacer molecules are incorporated into the perovskite lattice. Because these spacers are too large to fit within the octahedral [PbI_6_]^4–^ structure, they form an insulating layer that separates the inorganic structure into conducting layers, each n octahedra thick. This structure can be described by the general formula R_*m*_A_*n*–1_B_*n*_X_3*n*+1_ (*n* = 1, 2, 3 ··· ∞), where R is a divalent (*m* = 1) or monovalent (*m* = 2) organic spacer molecule, A is an organic or inorganic cation (e.g., CH_3_NH_3_+ (MA^+^), HC­(NH_2_)^2+^ (FA^+^), Cs^+^), B is an inorganic cation (e.g., Pb^2+^, Sn^2+^, Ge^2+^), and X is a halide anion (e.g., I^–^, Br^–^, Cl^–^). If the spacer molecules have two binding sites (*m* = 1), they can directly bridge two adjacent inorganic layers, forming a so-called Dion–Jacobson (DJ) structure. If the spacer molecules have only one binding site that can bind to the inorganic layer (*m* = 2), they form a double spacer layer separated by a van der Waals gap, referred to as a Ruddlesden–Popper (RP) structure.^8–10^

In 2017, Kanatzidis et al. introduced quasi-2D Pb-based perovskites in which not just the larger cation R but also the smaller cation A together fill the interlayer space in an alternating way.^11^ This structure is called an alternating cations in the interlayer (ACI) space-type perovskite and can be described by the general formula RA_*n*_B_*n*_X_3*n*+1_. Like the RP structure, these alternating cations form double spacer layers. However, the stacking of the octahedral layers is more similar to the DJ structure, where the inorganic layers are stacked perfectly on top of each other, lacking the half-unit cell shift in both in-plane directions present in the RP structure.^12,13^ Most ACI perovskites are formed by a combination of guanidinium (GA^+^, C­(NH_2_)^3+^) and methylammonium (MA^+^) cations and are described by the formula (GA)­(MA)_*n*_Pb_*n*_I_3*n*+1_. Various groups have studied quasi-2D ACI perovskite films based on the GA/MA cation combination. Similar to RP and DJ films, multiple domains of different *n*-phases are typically present within a film.^14–16^ Several strategies, including variations on spin-coating techniques,^15^ solvent^16^ and additive^14,17,18^ engineering, have been investigated to enhance material properties. These approaches aim to optimize the distribution and orientation of the different *n*-phases, improve the crystallinity and film smoothness, and thereby improve the optoelectronic properties, such as enhanced charge carrier transport and extraction and suppressed nonradiative charge recombination.

The GA^+^ cation is a particularly interesting spacer, as it is a resonance structure with the positive charge delocalized over the 3 NH_2_ groups, which might potentially enhance charge transfer between adjacent inorganic layers. The photophysics and photodynamics of ACI-type perovskites have not been widely explored yet. In some cases, transient absorption (TA) spectroscopy was employed mainly to study the spatial phase distribution in the layer. The variation in the differential absorption (Δ*A*) of the different *n*-phases with excitation from the front or backside indicates the spatial distribution of these phases. The conclusions from these studies are not consistent. On one hand, many authors propose electron transport from low-*n* to high-*n* domains accompanied by hole transfer in the opposite direction in (GA)­(MA)_*n*_Pb_*n*_I_3*n*+1_ films, based on TA data in which the low-*n* ground-state bleach (GSB) decay is accompanied by the formation of the *n* = ∞ GSB.^14,16^ On the other hand, Wang et al. studied GA­(MA)_3_Pb_3_I_10_ layers and interpreted the same observation as energy transfer instead of charge transfer.^17^ Photophysical modeling of the spectrotemporal TA data by global or target analysis has yet to be performed. Materny et al. studied the decay mechanisms in (GA)­(MA)_*n*_Pb_*n*_I_3*n*+1_ ACI perovskites with *n* = 1, 2, 3 extensively and observed that for low-*n*, exciton self-trapping is the dominant decay mechanism, while for increasing *n*-value, multiple exciton recombination (bi- or higher-order Auger recombination processes) becomes more dominant.^19^ However, in this study, low-*n* to high-*n* exciton or charge transfer was not considered.

In the present work, we report our study of the photodynamics of (GA)­(MA)_*n*_Pb_*n*_I_3*n*+1_ with ⟨*n*⟩ = 5. We steered the composition of the interlayer space via the GA:MA ratio in the precursor solution. During crystallization, the formation of the MA-based crystal lattice is energetically the most favorable and therefore occurs as the first step, since the GA^+^ spacer will encounter more steric hindrance than the MA^+^ spacer. Subsequently, the MA^+^ cations can occupy the interlayer space alongside the GA^+^ spacers. With a stoichiometric precursor solution, the leftover spacers that can form the interspace are GA:MA = 1:1 and alternate: GA-MA-GA-MA-GA-MA··· The structural formula can be rewritten as [(GA)­(MA)]­(MA)_*n*−1_Pb_*n*_I_3*n*+1_, where the term within square brackets indicates the cations in the interlayer space. If part of the GA^+^ cations in the solution are replaced by MA^+^, such that the ratio of these interlayer space cations will be GA:MA = 1:2, but the total amount of A-site cations in the solution remains the same, an interlayer might be formed with GA-MA-MA-GA-MA-MA-GA-MA-MA···, or vice versa (Figure 1).

**Figure 1:**
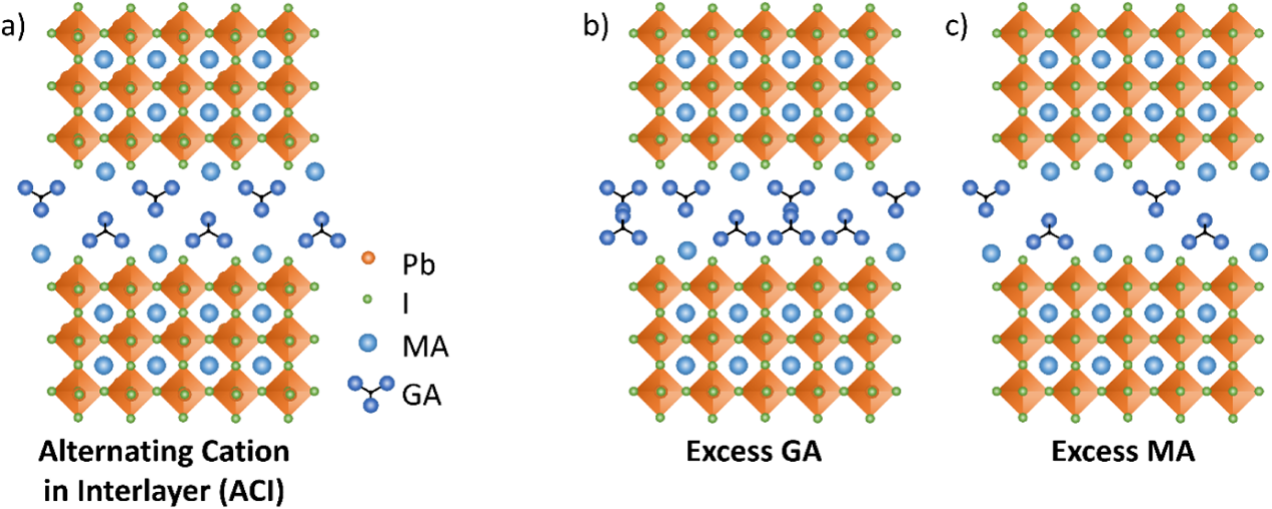
Schematic illustration of (a) the ACI perovskite, (b) the ACI perovskite with excess GA, and (c) the ACI perovskite with excess MA.

The aim of this study is to unravel the directionality, nature (i.e., excitons or charge carriers), and dynamics of transfer processes between the individual *n*-phases, and how these processes can be controlled by tuning the GA:MA ratio. We disentangle these transfer processes from the thermalization of hot carriers and/or excitons by femtosecond TA and time-resolved photoluminescence (TRPL) experiments, combined with photophysical modeling by target analysis. Our results provide a comprehensive understanding of the photophysics in these materials and their tunability via the GA:MA ratio, enabling the development of ACI perovskites for a new generation of PV devices.

## Experimental Section

### Materials

Lead­(II) iodide (PbI_2_, 99%), methylammonium iodide (MAI, ≥99%), *N*,*N*-Dimethylformamide (DMF, 99.8%), dimethyl sulfoxide (DMSO, ≥99.9%), and guanidinium iodide (GAI, ≥99%) were purchased from Sigma-Aldrich. These materials were used as received, without further purification.

### Preparation of Quasi-2D Perovskite [(GA)_1+*x*_(MA)_1–*x*_]­(MA)_*n*−1_(Pb)­n­(I)_3*n*+1_ Precursor Solutions

GAI, MAI, and PbI_2_ powders were dissolved in a stoichiometric molar ratio according to the *n*-value and the varying GA:MA ratio in the interlayer with a Pb^2+^ concentration of 0.6 M in the mixed solvent DMF:DMSO = 10:1 (v/v). This DMF:DMSO ratio was chosen as it yielded films with the highest XRD intensities, indicating the highest crystallinity.

### Fabrication of Quasi-2D Perovskite Thin Films

First, the ultraflat quartz-coated glass substrate was sequentially cleaned with a detergent, deionized water, acetone, and isopropyl alcohol in an ultrasonic bath. Then, the substrate was treated by O_2_ plasma. After this, the substrate was transferred to the N_2_-filled glovebox and attached to the chuck of the spin-coater, and 60 μL of precursor solution was dynamically spin-coated on the substrate with a stepwise spinning program of 1500 rpm for 15 s and 4000 rpm for 20 s. During the second step, 180 μL of chlorobenzene was dropped quickly onto the substrate. The final film was formed by postannealing at 100 °C on a hot plate for 15 min.

### Steady-State Characterization

X-ray Diffraction (XRD) patterns were measured by a Panalytical X’pert Pro Powder diffractometer with Cu Kα1 radiation. UV–vis transmittance spectra were recorded by using a Shimadzu UV-1800 spectrometer. During the measurements, the samples were contained in a quartz cuvette filled with nitrogen to prevent contact with moisture and oxygen. Scanning electron microscopy (SEM) images were obtained with a Zeiss Merlin HR-SEM.

### Time-Resolved Photoluminescence

Photoluminescence (PL) spectra and lifetimes with high time resolution were recorded with a Hamamatsu streak camera (C10910) equipped with a synchroscan sweep module (M10911-01). The samples remained in a nitrogen-filled quartz cuvette (Cotslab, QS38) throughout the process, from fabrication to measurement. The samples were excited using pulses with a center wavelength of 532 nm from a Fianium femtosecond laser with a pulse duration of 300 fs full width at half-maximum (FWHM) and a repetition rate of 80.37 MHz. A quartz lens with a 50 mm focal length was used to focus the laser beam onto the sample. The intensity of the excitation beam was kept below 5 mW at the sample position to avoid photobleaching. The UV–vis spectra before and after the TRPL measurements were verified to be identical. The emitted light was collected and focused on the input of the spectrograph (Acton SP2300, Princeton Instruments, using the grating with 50 lines/mm blazed at 600 nm) by two 2-inch diameter, 50 mm focal length glass lenses. A 570 nm long-pass filter was placed in front of the spectrograph to block the scattered laser light and protect the device from potential damage. The output of the spectrograph was sent to the photocathode of the streak camera. To correct for the spectral sensitivity of the setup, a spectral sensitivity correction was performed based on the measured and provided emission spectrum of a blackbody calibration lamp (Ocean Optics, HL-2000). Background correction was performed by subtracting the average of the spectra prior to *t* = 0 from the dataset, i.e., a constant value was subtracted for each PL wavelength. A long-lived high-*n* PL signal, present as a back-sweep signal before *t* = 0 in the streak camera image, was subtracted from the high-*n* PL data as a background signal. This correction affects the intensities of the low-*n* PL bands relative to those of the high-*n* PL bands but does not have any influence on the PL dynamics relevant for photophysical modeling and the resulting rate constants.

### Time-Correlated Single-Photon Counting (TCSPC)

To measure PL lifetimes beyond the 1–2 ns time window accessible by the streak camera, a TCSPC laser scanning confocal microscope (PicoQuant, MT200) was used. The samples remained in a nitrogen-filled cuvette from fabrication in the glovebox to the microscopy studies. A pulsed laser source with a repetition rate of 2 MHz (PicoQuant, LDH-D-C-485), an excitation wavelength of 485 nm, and a pulse duration of ∼100 ps FWHM was used for photoexcitation. The laser light was directed toward the sample using a dichroic mirror (Chroma, ZT405/488rpc-UF3). The sample was illuminated through a 20× objective (Olympus, LUCPlanFL N 20×) with a power density of approximately 1 W/cm^2^, and the PL was collected by the objective and directed through a pinhole toward three single photon avalanche detectors (SPADs, Excelitas, SPCM-AQRH-14-TR). Each SPAD was set to a specific spectral region. The initial attempt to detect using the spectral regions with a 520/35 nm green band-pass and a 620/60 nm orange band-pass did not yield significant intensity. Therefore, the lifetime analysis was based on the red channel, using a long-pass 633 nm filter (RazorEdge LP Edge Filter 633 RU). The lifetime histograms were processed using a customized Python script that fits the histograms to a fourth-order exponential model using a nonlinear least-squares minimization method. The script then calculated the intensity-weighted average lifetime from the fitted model.

### Transient Absorption

The fs TA setup was described in detail in our earlier work.^20^ In short, the system included a Coherent Micra seed laser that generated 800 nm pulses with a pulse duration of 35 ± 1 fs (FWHM) at an 80 MHz repetition rate. These pulses were amplified to 800 nm pulses at a 5 kHz repetition rate using a Coherent Legend Ti:sapphire amplifier. The output was split into pump and probe beams with an 85:15 beamsplitter. The pump beam was sent through an optical parametric amplifier system (TOPAS-Prime with NIRUVIS extension, Light Conversion) to generate 490 nm pulses and chopped to 2.5 kHz to generate a “pump on” and “pump off” mode to determine the differential absorbance (Δ*A*). The probe beam was guided through a mechanical delay stage and subsequently focused into a CaF_2_ crystal (Newlight Photonics, 3 mm thickness) to generate a white light continuum probe. The CaF_2_ crystal was mounted on a motorized translational stage and moved with ca. 2 mm/s to avoid thermal damage, yielding a stable continuum from ca. 350–825 nm. The remaining fundamental was attenuated using NENIR30 and NENIR20 filters from Thorlabs. The polarization of the probe beam relative to that of the pump beam was set at 54.7° (magic angle) to avoid anisotropy effects.^21^ The pump and probe beams were focused and spatially overlapped at the sample with spots of approximately 250 and 50 μm diameter, respectively. The transmitted pump beam was blocked, and the transmitted probe beam was directed toward a home-built detector system consisting of a 15 cm spectrograph (Acton SP-150, 150 lines/mm grating with a 300 nm blaze set at a center wavelength of 550 nm) and a 256-pixel diode array detector. The samples were placed in a quartz cuvette with the lid sealed with parafilm and never in contact with oxygen or moisture, still within the nitrogen environment of the glovebox. We observed earlier that such sealing excludes any O_2_-induced reduction in the phosphorescence lifetime of Ru-polypyridyl complexes dissolved in acetonitrile and purged with N_2_ for at least 1 day.^22^ During the measurement, the samples were mounted on a continuously moving translational stage to prevent photobleaching, and the TA signal was verified to remain similar during the experiment, excluding significant photoinduced sample changes. As our focus is on ultrafast processes, the early time delay steps were set to small values (20 fs up to 2.5 ps and 30 fs up to 5 ps) and gradually increased with delay time. Although the white light continuum is stable, strong light absorption by the sample implies that fewer probe photons are capable of reaching the detector, lowering the signal-to-noise ratio. As a result, the signal-to-noise ratio depends on the probe wavelength and is the best in the case of weak absorption by the sample. Therefore, TA measurements at shorter wavelengths were not possible, as the absorbance of our samples was very high in that wavelength region. In the case of the TA measurements with 490 nm excitation, a few more photons in the short wavelength range could reach the detector due to slightly thinner samples, enabling a broader wavelength region to be measured. We decided not to fabricate thinner layers, as this would lower the TA and PL signals and therefore require higher pump intensities, leading to photobleaching and second-order photophysical processes, and influence the crystallization and distribution of *n*-phases. The pump power was kept relatively low, with energy densities between 5 and 15 μJ/cm^2^ and verified to be in the linear regime, where the intensity of the TA signal increases linearly with the incident pump intensity. The TA spectra were analyzed by target analysis in the open-source program Glotaran.^23^

## Results and Discussion

### Steady-State Characterization

Figure 2 shows the steady-state UV–vis absorption spectra of the films with varying GA:MA ratios. The signal in the IR (around 800 nm) is due to reflection and scattering. For all films, an absorption onset characteristic of the 3D phase (*n* = ∞) is evident, with absorption onsets ranging from 1.573 eV (788 nm, 2GA-MA) to 1.596 eV (777 nm, GA-2MA), as determined from Tauc plots (Figure S1). The latter value is consistent with values reported in the literature.^6^ Furthermore, excitonic absorption bands centered at approximately 586 nm (*n* = 2), 621 nm (*n* = 3), 652 nm (*n* = 4), and 678 nm (*n* = 5) are observed. These bands are most prominent for the films with excess GA. In addition, the films have also been analyzed by XRD, showing peaks at about 14.1° and 28.3° (Figure S2). The surfaces of the films have been characterized by SEM (Figures S3–S5).

**Figure 2:**
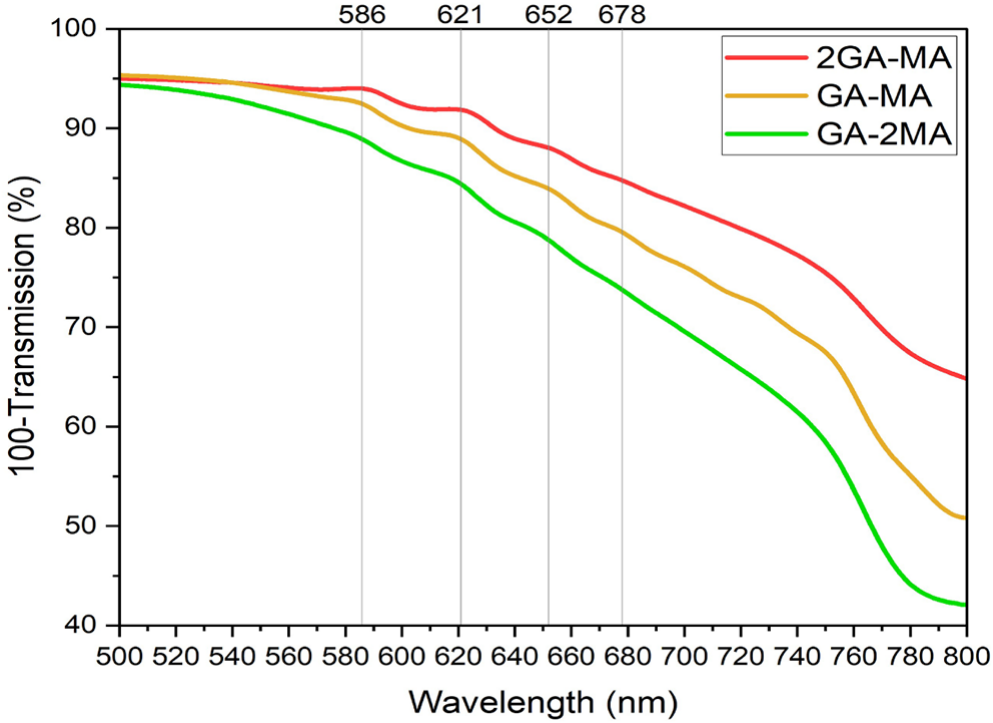
Steady-state UV–vis absorption spectra of the GA-MA layers with varying GA:MA ratios. The excitonic bands around 586 nm (*n* = 2), 621 nm (*n* = 3), 652 nm (*n* = 4), and 678 nm (*n* = 5) are indicated with gray lines.

### Time-Resolved Photoluminescence

Figure 3 presents the TRPL spectra following 532 nm back-side excitation of the films (i.e., the light is incident on the quartz substrate). With front-side illumination (i.e., the light is incident on the perovskite film), only one PL band centered around 765 nm and extending up to ∼825 nm is observed, originating from the high-*n* phase (Figure S6). This observation indicates that high-*n* domains are dominant near the exposed perovskite surface, as also observed in other studies.^14,16^ Note that the high-*n* emission maximum observed in the TRPL spectra is slightly blue-shifted relative to the absorption onset (Figure 2), the effect of which is likely due to the pulsed illumination in the first experiments. Loi et al. found a 75 meV PL blue-shift with increasing excitation power and assigned this observation to state-filling of band edge states.^24^ In contrast to front-side illumination, with back-side illumination, both low-*n* and high-*n* PL bands are observed, indicating that the lower-*n* domains are located nearer to the substrate side of the films. The PL bands are centered around 595 nm (*n* = 2), 630 nm (*n* = 3), and 700 nm (*n* = 4, 5), with some variations in wavelengths and relative intensities for the different GA:MA ratios. The Stokes shift for *n* = 2 of ca. 9 nm is significantly smaller than the 25 nm observed earlier for propylammonium (PA), 1,4-phenylene dimethylammonium (PDMA), and PDMA–PA mixed spacer films,^25^ suggesting less exciton–phonon coupling.^26,27^ For the photophysical modeling below, we defined the PL band centered at 595 nm as low-*n*, around 630 nm as high-low-*n*, the PL ∼640–700 nm as intermediate-*n*, and around 765 nm and extending up to ∼825 nm as high-*n*. Note that the PL spectra are cut off by a 570 nm long-pass filter, thereby not fully resolving the PL band centered around 595 nm. The PL spectra exhibit a slight negative signal >825 nm after 400 ps. This is an artifact of the background correction, as the PL signal in this region is very weak, causing the slightly uneven background to influence the results. The low-*n* PL signals are short-lived, in the order of the TRPL instrumental response time (IRT, ∼16 ps), indicative of PL quenching. These short-lived lower-*n* PL bands are especially observed for the films with excess GA and not as clear in the GA-2MA films, as could be anticipated from the UV–vis spectra (Figure 2) showing less distinct lower-*n* excitonic bands for the latter.

**Figure 3:**
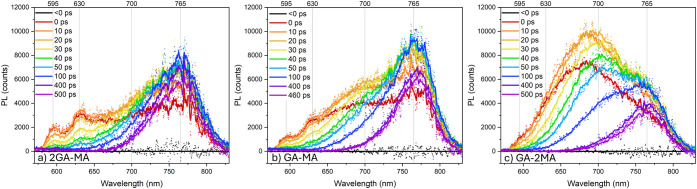
TRPL spectra following 532 nm back-side excitation measured by streak camera detection of (a) 2GA-MA, (b) GA-MA, and (c) GA-2MA films recorded in reflection mode. The solid lines present fits from the photophysical modeling. The gray lines indicate the PL bands from *n* = 2, *n* = 3, *n* = 4, 5, and *n* = ∞ phases. Note that the spectra are cut off due to the use of a 570 nm long-pass filter.

Figure 4 displays the normalized TRPL kinetic traces at key wavelengths. Most traces develop fully within the IRT following excitation and subsequent decay, with the decay becoming slower with increasing wavelength. The low-*n* PL mainly decays within the IRT (ca. 90%, see Figure S7) with only a weak longer-lived component. This observation suggests the presence of one dominant low-*n* species, in contrast to our recent findings on RP and DJ quasi-2D perovskites, where two distinct low-*n* species were observed (∼70% with a fast PL decay, ∼30% with a slow PL decay) with almost similar species-associated spectra (SAS).^25^ We assigned the former to excitons within the exciton diffusion length from the domain edge, which are transferred into high-*n* domains resulting in PL quenching, and the latter to excitons in the middle of low-*n* domains not transferred into high-*n* domains. For the 2GA-MA and GA-MA films, the high-*n* PL signal develops slightly more slowly than the IRT, while this is not the case for the GA-2MA film. Analogous to our recent work, we assign this observation to ∼10 ps exciton transfer from low-*n* into higher-*n* domains,^25^ occurring slightly slower in the film with excess GA. Furthermore, only for the 2GA-MA film does the high-*n* PL develop slightly further until ∼200 ps. Considering the higher absorption between 600 and 700 nm with increasing amounts of GA (Figure 2), this observation suggests minor reabsorption of the low-*n*, high-low-*n*, and/or intermediate-*n* PL by the high-*n* domains. The PL decays of the high-low-*n* and intermediate-*n* domains do not lead to a synchronous further increase in high-*n* PL, indicating charge transfer rather than exciton transfer to high-*n* domains. This interpretation is consistent with the high exciton binding energy in low-*n* domains (a few tens of meV)^28,29^ and the low exciton binding energy in higher-*n* domains.^29,30^ Excitons in the latter domains are therefore assumed to dissociate. The PL decay of the intermediate-*n* domains (∼640–700 nm), indicative of charge transfer into the high-*n* domains, is the slowest for the 2GA-MA film. The charge carriers in the high-*n* domains decay on a ns time scale, as follows from TCSPC, with the longest lifetime for the GA-2MA films (Figure S16), suggesting fewer defects in the high-*n* domains.

**Figure 4:**
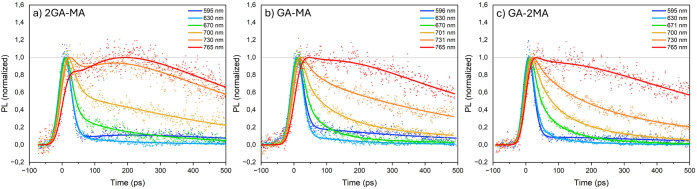
TRPL kinetic traces at selected wavelengths following 532 nm back-side excitation measured by streak camera detection of the (a) 2GA-MA, (b) GA-MA, and (c) GA-2MA films recorded in reflection mode. The solid lines indicate fits from the photophysical modeling.

### Transient Absorption

To develop further mechanistic insight into the photophysics of the materials, fs TA measurements have been performed using 630 and 490 nm excitation wavelengths. Additionally, potential accumulation effects are expected to be negligible here, as we photoexcite with a low repetition rate of 2.5 kHz. The 630 nm excitation yields less complex dynamics, as the photon energy is insufficient to excite the low-*n* phase, in contrast to the 490 nm excitation. Using both excitation wavelengths enables one to disentangle exciton transfer from the low-*n* phase to the high-low-*n* phase from charge transfer from the latter into the intermediate-*n* and the high-*n* domains and the thermalization of hot excitons and charge carriers. The films have been photoexcited from the backside, as, according to the TRPL data, this side contains the highest fraction of low-*n* domains, maximizing the occurrence of exciton and charge transfer between the domains.

Figure 5 shows the TA spectra of the various films following 630 nm excitation. Because of the pump spectrum, the TA spectra are shown above 660 nm. The broad negative signal >660 nm is due to ground state bleach (GSB) of the intermediate-*n* and high-*n* phases. Above 780 nm, photoinduced absorption (PIA) is observed.^31^ The kinetic traces presented in Figure 6 show that the GSB has a sub-ps initial decay indicative of hot carrier thermalization,^32,33^ followed by a slower decay, which is slower at higher wavelengths (high-*n*) compared to the lower wavelengths (intermediate-*n*). Interestingly, for all films, the kinetic traces at higher wavelengths, e.g., around 730 nm, exhibit a ∼100 ps growth, while in the TRPL, this phenomenon is only observed for the 2GA-MA film. The latter was assigned above to minor PL reabsorption, which is most likely in the 2GA-MA film because of its high absorption from 600 to 700 nm. As we do not observe a ∼100 ps rise in the high-*n* TRPL traces of the other films, we assign this TA feature to charge transfer from intermediate-*n* into high-*n* domains. The high-*n* GSB decays due to charge recombination.

**Figure 5:**
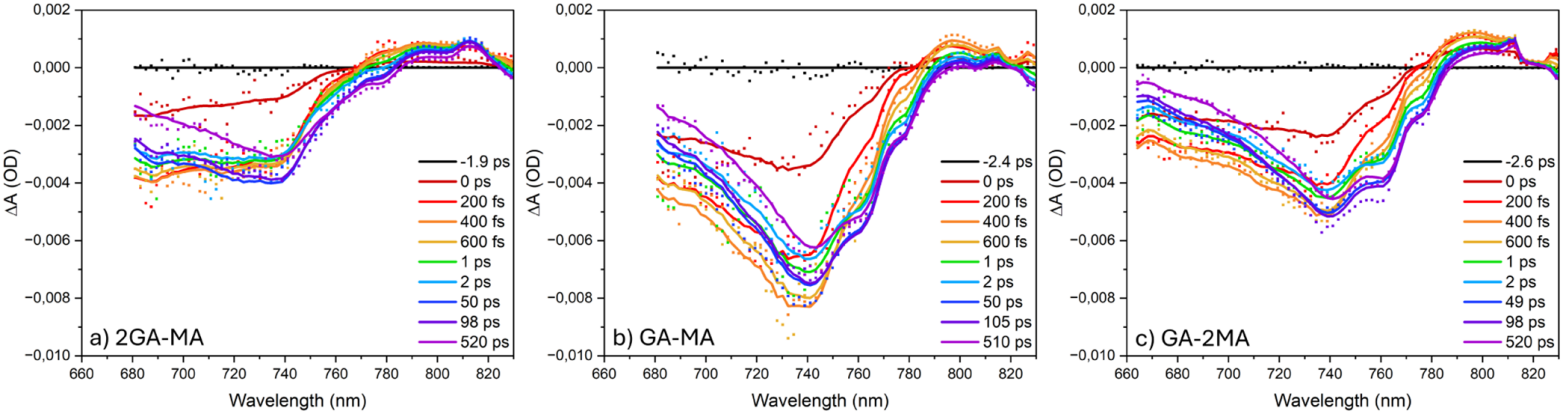
TA spectra using 630 nm excitation from the back-side of the (a) 2GA-MA, (b) GA-MA, and (c) GA-2MA films. The solid lines indicate fits from photophysical modeling.

**Figure 6:**
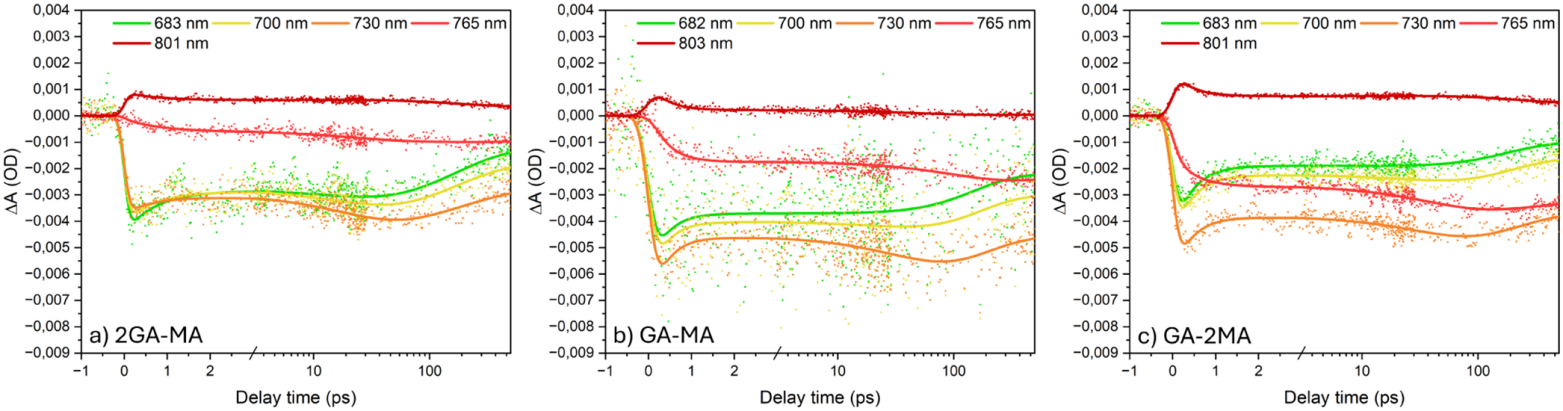
TA kinetic traces at various wavelengths using 630 nm excitation from the back-side of the (a) 2GA-MA, (b) GA-MA, and (c) GA-2MA films. The solid lines indicate fits from photophysical modeling.

The TA spectra at 490 nm excitation, shown in Figure 7, exhibit additional GSB bands centered at approximately 586 nm (*n* = 2), 621 nm (*n* = 3), and 652 nm (*n* = 4), with the fastest decay for the lowest wavelengths. For all films, the high-*n* GSB red-shifts within ∼1 ps, indicating hot carrier thermalization.^32,33^ Interestingly, the kinetic traces presented in Figure 8 show that after the subpicosecond initial decay, especially the ∼586 nm band in the 2GA-MA and GA-MA films exhibits a ∼10 ps decay, indicative of exciton transfer from low-*n* into high-low-*n* domains also indicated by TRPL. This decay is less clear in the TA data with excess MA, consistent with the less distinct excitonic bands (Figure 2) and weak low-*n* PL (Figure 3). Alike for the TA data obtained using 630 nm excitation, the ∼100 ps high-*n* GSB growth is again observed and assigned to charge transfer from high-low-*n* to intermediate-*n* and from there into high-*n* domains.

**Figure 7:**
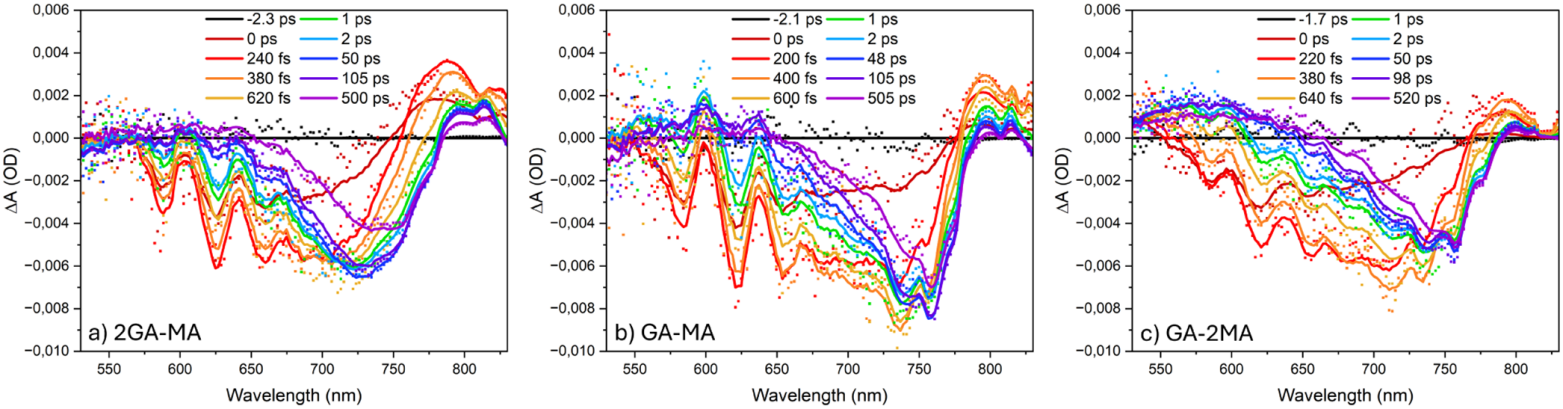
TA spectra using 490 nm excitation from the back-side of the (a) 2GA-MA, (b) GA-MA, and (c) GA-2MA films. The solid lines indicate fits from photophysical modeling.

**Figure 8:**
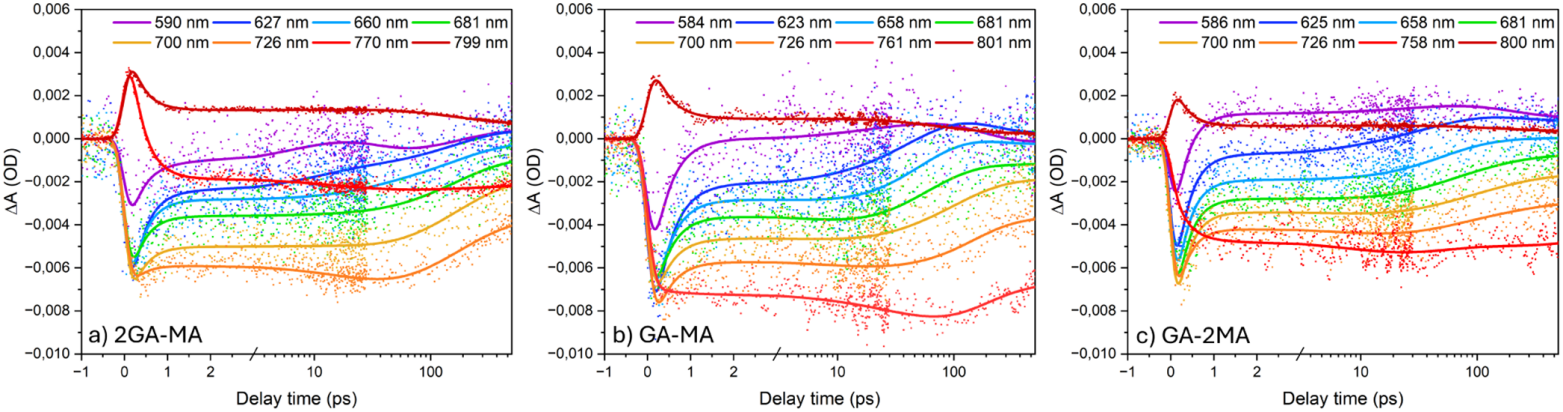
TA kinetic traces at various wavelengths using 490 nm excitation from the back-side of the (a) 2GA-MA, (b) GA-MA, and (c) GA-2MA films. The solid lines indicate fits from photophysical modeling.

### Photophysical Modeling

To quantify the dynamics of these photophysical processes, target analysis has been performed by applying a unified model to all data sets to ensure consistency (Figure 9). The model assumes that hot excitons and charge carriers are generated within the TA instrument’s response time (IRT, 100–150 fs). To avoid overmodeling, these are considered as a single hot species, thermalizing with a decay rate *k*_1_ into the low-*n*, high-low-*n*, intermediate-*n*, and high-*n* phases. This assumption is valid since hot carrier cooling generally occurs in <1 ps,^34,35^ and vibrational relaxation of hot excitons also takes place in ∼1 ps.^36,37^ Thermalization is followed by a stepwise exciton (low-*n* into high-low-*n*) or charge transfer (high-low-*n* to intermediate-*n* and from there into high-*n*), with *k*_*x*_ denoting the rate constant of the individual process. Low-*n* excitons are transferred into high-low-*n* domains with *k*_2_, excitons in the latter domains have a lower exciton binding energy than in the former and are therefore assumed to dissociate.^28–30^ High-low-*n* charge carriers are transferred into intermediate-*n* domains with *k*_3_, and from there into the high-*n* phase with *k*_4_. Finally, high-*n* charge carriers recombine with the rate constant *k*_5_. To avoid overmodeling, PL reabsorption that may occur in the 2GA-MA films on a ∼100 ps time scale has not been included. This assumption is justified since the TRPL data of the 2GA-MA film (Figure 4a) indicate only minor reabsorption, while the effect is even absent in the TRPL data of the other layers (Figure 4a,c).

**Figure 9:**
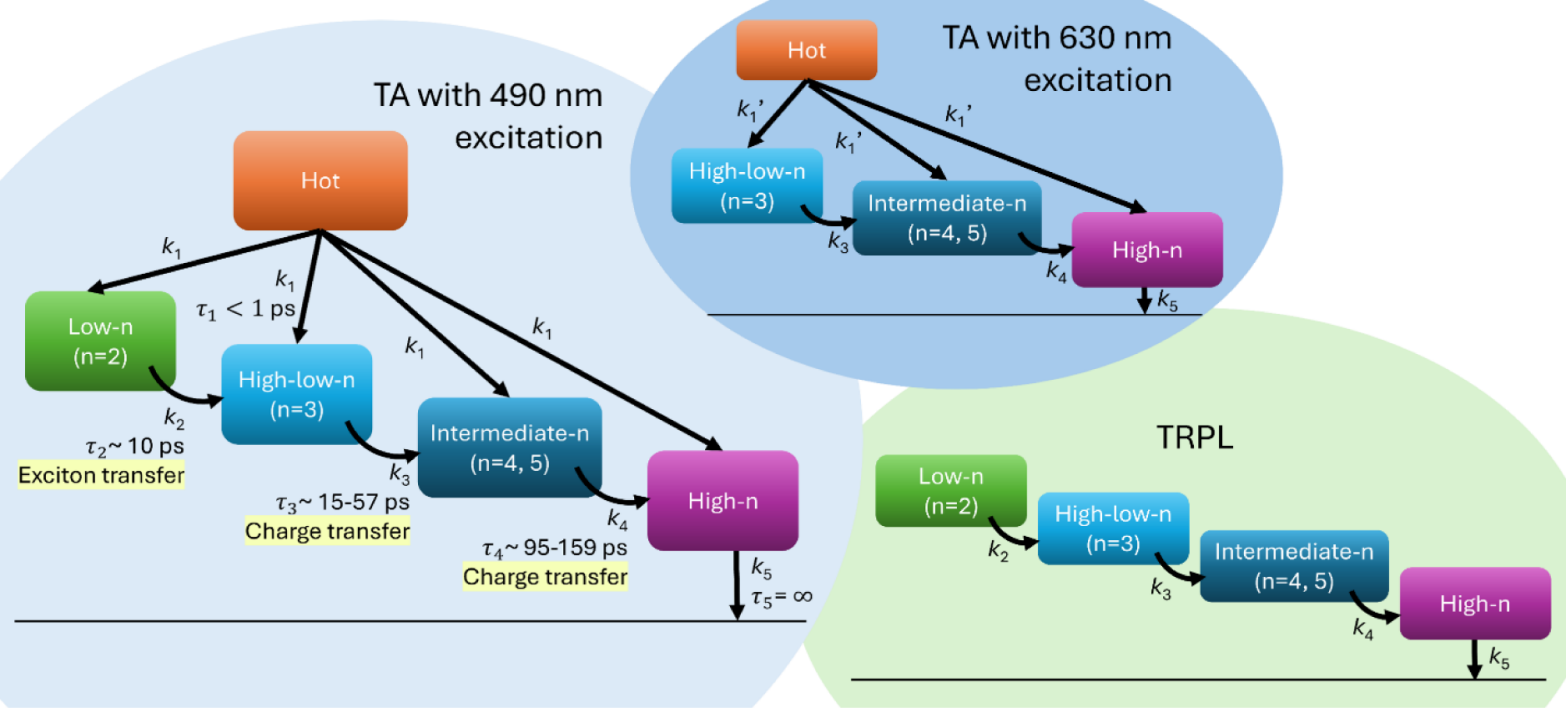
Photophysical model used for target analysis of the TA data at 490 nm excitation and the adjusted models applied to the TA data at 630 nm excitation and the TRPL data. The symbol ′ indicates the need for rescaling of the *k*_1_ values, as the low-*n* phase is not excited at 630 nm.

In modeling the TRPL data, it is assumed that the initially generated hot excitons and charge carriers have already cooled during the instrument response time (IRT, ∼16 ps). This assumption is valid since hot carrier cooling is generally found to occur within 1 ps,^34,35^ and vibrational relaxation of hot excitons also occurs in ∼1 ps.^36,37^ As a boundary condition, the requirement that the PL spectra obtained from modeling should be positive was set. Furthermore, a sequential model, rather than a parallel model, is needed to model the wavelength-dependent ingrowth in the TRPL signal (Figure 4). We observed that at least three components are needed to describe the TRPL data. However, as modeling the TA data requires a fourth cooled species, the TRPL data have also been modeled using a sequential model with four components (Figure 9). Resolving this extra component was challenging without fixing some of the other decay rates, especially for the 2GA-MA and GA-MA films. Hence, for these films, the TRPL was first modeled using 3 components. The TRPL decay traces show that the initial decay is within the IRT for all the films, making accurate estimation of the fast decay rate constant *k*_2_ through fitting impossible. To achieve accurate fits for intermediate-*n* PL decay rate *k*_4_, the value of *k*_2_ was therefore set to 0.1 ps^–1^. This value was determined after testing several values, and we concluded that 0.1 ps^–1^ was the smallest rate constant that could adequately model all of the rapid low-*n* PL decays. Hence, 0.1 ps^–1^ serves as a lower bound for the actual *k*_2_ value. With modeling of the TRPL data, *k*_4_ was obtained. Note that the values of *k*_5_ could not be accurately determined from the TRPL data due to the limited experimental time window and the presence of a back-sweep signal, which removes the long component after background correction. Hence, the *k*_5_ values obtained from the TRPL data were not used for further analysis. Instead, *k*_5_ was obtained from time-correlated single-photon counting (TCSPC) measurements (Figure S16).

In the TA experiments with excitation at 630 nm, the low-*n* phase is not excited and is therefore excluded from the model. For fitting the TA data with 630 nm excitation, *k*_5_ and the *k*_4_ value obtained from TRPL were used as input. The values of *k*_1_ and *k*_3_ were determined by fitting. Interestingly, the obtained τ_3_ (=1/*k*_3_) value was in the range of 21–54 ps, which is slower than the ≤ 10 ps low-*n* component of the TRPL. This observation confirms that the high-low-*n* species is not simply the same as the low-*n* species but a component that can actually be observed separately. Besides, fixing *k*_3_ to 0.1 ps^–1^ resulted in worse fits. The *k*_1_ and *k*_3_ values obtained from fitting the TA data with 630 nm excitation were used as input for modeling the TA data with 490 nm excitation together with the previously determined *k*_2_, *k*_4_, and *k*_5_. As the *k*_2_ values in TRPL are within the IRT, *k*_2_ = 0.1 ps^–1^ is likely an underestimation. Therefore, it would be more accurate to obtain *k*_2_ from the TA data. However, when *k*_2_ was not fixed while modeling the TA data with 490 nm excitation, the fitting produced poor results, as *k*_2_ was estimated to be an unrealistically high value. Therefore, *k*_2_ was also fixed to 0.1 ps^–1^ in the modeling of the 490 nm excitation TA data. When *k*_1_ was fixed to the value obtained by fitting the TA data with 630 nm excitation, the resulting fitted initial decays were noticeably too slow. However, the model did not produce results when *k*_1_ was left as a free parameter. Hence, *k*_1_ was determined by separately fitting the positive feature around 800 nm and then fixed in the model for modeling the entire data set. With all *k*-values fixed, the amplitudes of the various components, the IRT, and the dispersion were obtained. Finally, the *k*_3_ value obtained from fitting the TA data with 630 nm excitation was incorporated into the TRPL model, and the 4-component model was applied to the TRPL data with *k*_2_, *k*_3_, and *k*_4_ held fixed. This procedure allowed for the evaluation of the fits and species-associated spectra (SAS) to determine whether the addition of this component could be validated. The models describe the TRPL and TA data well, as can be observed in Figures 3–8. Table 1 presents the time constants obtained by modeling the TA data at 490 nm excitation; those from fitting the TRPL data with 3 and 4 parameters and the TA data at 630 nm excitation are presented in Tables S1–S3.

**Table 1:** Time Constants (*k*) and Lifetimes (τ) from Target Analysis of the TA Data under 490 nm Excitation for the Films with Varying GA:MA Ratios, Based on the Model Shown in Figure 9 ^a^

Film	*k*_1_ [ps^–1^]	*k*_2_ [ps^–1^]	*k*_3_ [ps^–1^]	*k*_4_ [ps^–1^]	*k*_5_ [ps^–1^]
2GA-MA	0.767 (f^b^)	0.1 (f^*^ ^c^)	4.6 × 10^–2^ (f^d^)	6.3 × 10^–3^ (f^c^)	1.42 × 10^–4^ (f^e^)
GA-MA	0.618 (f^b^)	0.1 (f^*^ ^c^)	1.9 × 10^–2^ (f^d^)	1.0 × 10^–2^ (f^c^)	1.13 × 10^–4^ (f^e^)
GA-2MA	0.801 (f^b^)	0.1 (f^*^ ^c^)	2.3 × 10^–2^ (f^d^)	7.1 × 10^–2^ (f^c^)	1.08 × 10^–4^ (f^e^)
	τ_1_ [ps]	τ_2_ [ps]	τ_3_ [ps]	τ_4_ [ps]	τ_5_ [ps]
2GA-MA	326 × 10^–3^	10 (±−)	21.6 (±0.7^d^)	158.8 (±1.6^c^)	7.050 × 10^3^ (±0.38^e^)
GA-MA	404 × 10^–3^	10 (±−)	53.5 (±5.4^d^)	95.4 (±5.1^c^)	8.813 × 10^3^ (±0.18^e^)
GA-2MA	312 × 10^–3^	10 (±−)	43.7 (±0.7^d^)	140.0 (±2.3^c^)	9.278 × 10^3^ (±0.15^e^)

a Note that $\tau_{1}=\frac{1}{4*k_{1}}$ because of the four cooling pathways of the hot carriers and excitons.

b Determined from separately fitting the PIA around 800 nm.

c Determined from the TRPL data.

d Determined from the TA data with 630 nm excitation.

e Determined from the TCSPC data.

The hot carrier cooling times (τ_1_) obtained from the TA data with 490 nm excitation are 0.3–0.4 ps, consistent with earlier studies describing sub-ps cooling of hot carriers^34,35^ and excitons.^36,37^ The high-low-*n* to intermediate-*n* charge transfer time (τ_3_) is in the range of about 22–54 ps and is the fastest for the 2GA-MA films. Work by Materny et al. on ACI perovskites reports an increase in barrier height with *n*-value for self-trapped exciton (STE) formation, resulting in dominant STE formation, especially in lower-*n* layers.^19^ We cautiously assigned the short τ_3_ value for the 2GA-MA layers to the fastest STE formation. Competition of charge transfer into intermediate-*n* domains with fast STE formation in high-low-*n* domains could also explain why high-low-*n* PL bands around 630 nm are especially observed with excess GA and less with excess MA (Figure 3). Analogous to earlier studies,^19,38–40^ we therefore assign the PL band centered at 630 nm to a combination of weakly bound electron–hole pairs and STEs. The 2GA-MA layers also show the slowest intermediate-*n* to high-*n* charge transfer time (τ_4_), which we cautiously assign to a relatively weak electronic coupling between these domains induced by the excess GA. The high-*n* lifetimes (τ_5_) determined by TCSPC are in the range of 7.1–9.3 ns, which are weighted averages of four components with lifetimes of 0.9–1.8 ns, 5.3–6.7 ns, 16–24 ns, and 47–89 ns. Consistent with the trend in the TCSPC data, τ_5_ increases with the MA quantity. In the GA-MA and GA-2MA films, the emitting material is uniformly distributed across the layer, in contrast to the 2GA-MA films showing regions with minimal PL (Figures S11–S13). These observations suggest that high-*n* domains in the MA-GA and especially the 2MA-GA layers contain fewer defects relative to layers with excess GA.

The present work is, to our best of our knowledge, the first to disentangle exciton and charge transfer from low-*n* domains toward higher-*n* domains. Earlier work by Lin et al. on reports sequential exciton transfer occurring in a time window of ∼2–300 ps in RP-type quasi-2D perovskites based on PEA with ⟨*n*⟩ = 3 (PEA = C_6_H_5_(CH_2_)_2_NH_3_).^34^ Similarly, studies on RP- and DJ-type quasi-2D perovskites report energy transfer from low-*n* toward higher-*n* phases.^41,42^ We found in our recent studies on PDMA-MA DJ^20^ and PDMA-PA mixed spacer DJ/RP^25^ systems exciton transfer to occur in a few tens of ps. As a next step, the exciton binding energy in lower-*n* domains in ACI perovskites should be compared to those in the DJ and RP systems.

Ideally, for PV applications, the charge carriers in the high-*n* domains are long-lived, and exciton or charge transfer from low-*n* to higher-*n* domains occurs fast, enabling the charge carriers to reach the contacts before recombination. Exciton transfer from low-*n* into high-low-*n* occurs for all layers studied within 10 ps and likely even faster. Consequently, we could not disentangle it from hot carrier and exciton cooling, and it could well be that these processes all occur in the same subpicosecond time window. At early times beyond (∼10–20 ps), films with excess GA show more high-low-*n* PL relative to the other layers, potentially caused by fast STE formation competing with charge transfer toward intermediate-*n* domains, negatively affecting the charge transfer yield. The subsequent charge transfer from intermediate-*n* to high-*n* domains occurs the slowest in layers with excess GA (∼159 ps) and the fastest for the MA-GA layers (∼95 ps), which is advantageous. The high-*n* lifetime (τ_5_) is the longest for the 2MA-GA and GA-MA layers and significantly shorter with excess MA. Notably, the former films also show more uniformly distributed PL and fewer areas with minimal emission (Figures S8–S10). These differences indicate fewer defects in the high-*n* domains of the 2MA-GA and GA-MA layers, likely favorable for charge transport and reducing recombination losses. The photophysical dynamics of the latter layers compared with those with excess GA can be expected to be beneficial for PV applications.

## Conclusions

This work focuses on ACI-type quasi-2D perovskites based on the guanidinium (GA) spacer and explores how changing the GA:MA ratio in the interlayer space for a ⟨*n*⟩ = 5 film influences the photophysical processes. Upon photoexcitation of all phases in these films, hot carriers are initially generated in the high-*n* domains, while nonthermalized excitons are formed in the low-*n* domains. After subpicosecond thermalization, excitons are transferred within 10 ps from the low-*n* to high-low-*n* domains, in which they dissociate into free charges. From there, charge transfer into the intermediate-*n* domains occurs in 22–54 ps. In the layers with excess GA, this process might occur in an undesirable competition with STE formation. From the intermediate-*n* domains, charge carriers are transferred into the high-*n* domains in ∼95–159 ps, which process occurs the fastest in GA-MA films. Finally, charge carriers decay intrinsically on the nanosecond scale, with the longest lifetimes for the GA-MA and GA-2MA systems, which is beneficial for PV applications.

## Supplementary Material



## Notes

Tauc plots; X-ray diffraction (XRD) patterns; scanning electron microscopy (SEM) images; TRPL spectra front-side excitation; TRPL time traces per wavelength; species associated spectra (SAS); time constants; time-correlated single photon counting (TCSPC) data (PDF)
