# Supplementary material for: Exploring the Effects of the Guanidinium:Methylammonium Ratio on the Photophysical Dynamics of ⟨n⟩ = 5 ACI Perovskites
Source: J Phys Chem C Nanomater Interfaces. 2025 Oct 1;129(40):18118–27. doi: 10.1021/acs.jpcc.5c04999 (PMC12516724; doi:10.1021/acs.jpcc.5c04999)
Supplement: Supplementary file 1 [file jp5c04999_si_001.pdf]

Supporting Information

Exploring the Effects of the Guanidinium: Methylammonium Ratio on the Photophysical Dynamics of ⟨n⟩=5 ACI Perovskites

Lisanne M. Einhaus,[†] Xiao Zhang,[‡] Jeroen P. Korterik,[¶] Robert Molenaar,[§] Guido Mul,[†] Johan E. ten Elshof,[‡] and Annemarie Huijser[*,†]

†PhotoCatalytic Synthesis Group, MESA+ Institute for Nanotechnology, University of Twente, 7500 AE, Enschede, the Netherlands

‡Inorganic Materials Science Group, MESA+ Institute for Nanotechnology, University of Twente, 7500 AE, Enschede, the Netherlands

¶Optical Sciences Group, MESA+ Institute for Nanotechnology, University of Twente, 7500 AE, Enschede, the Netherlands

§NanoBioPhysics Group, MESA+ Institute for Nanotechnology, University of Twente, 7500 AE, Enschede, the Netherlands

E-mail: j.m.huijser@utwente.nl

Supplementary Information

Tauc Plot

Tauc plots based on the UV-Vis spectra in the main text (Figure 2) are presented in Figure S1. The absorbance was calculated by $\alpha = -log(T)$, where T is the transmittance. Note that we do not take reflection into account in this calculation. Energy values were calculated using $E = hc/\lambda$, with h Planck's constant, c the speed of light, and λ wavelength. Subsequently, $(\alpha h\nu)^2 = (2.303 * E * \alpha)^2$. A linear line was manually fitted to the steepest part of the low-energy range. Horizontal lines are drawn fitting the horizontal part of the curve <1.55 eV. The x-value of the intersection of these two lines determines the estimated bandgap. The resulting bandgaps are in between 1.573 eV (2GA-MA) and 1.596 eV (GA-2MA) (i.e. between 788 and 777 nm). The Tauc plot for the 2GA-MA and GA-MA films do not have a horizontal part <1.55 eV. Therefore, the absorption onset cannot be accurately determined using this method. This might be caused by reflection effects. The original UV-Vis spectra show a clear red-shift in the absorption onsets as the relative amount of GA increases.

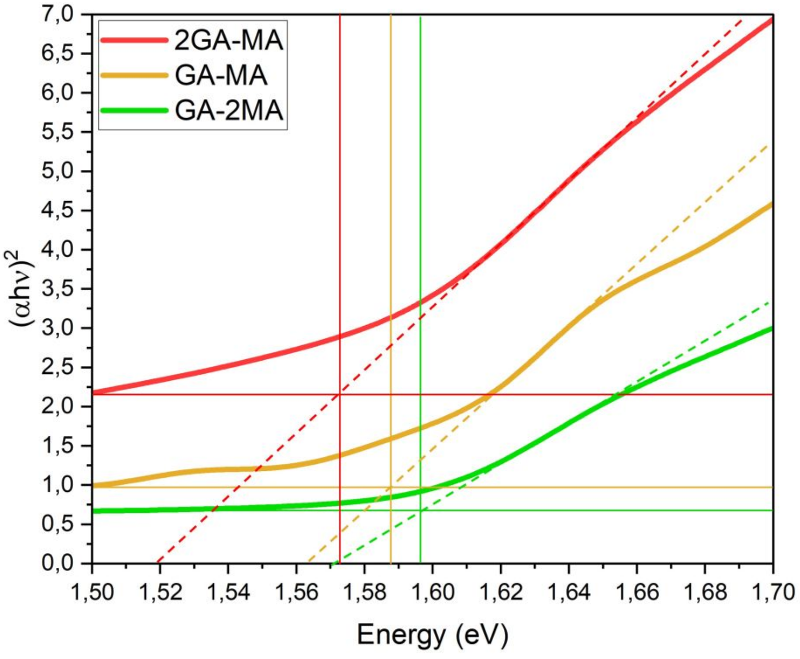

Figure S1: Tauc plots based on the UV-VIS data presented in Figure 2 of the main text.

X-ray Diffraction (XRD)

Figure S2 displays the X-ray diffraction (XRD) pattern of perovskite films with varying GA:MA ratio. The XRD peak at around 14.1° corresponds to the (111) lattice plane of the tetragonal $MAPbI_3$ phase. Similarly, the peak around 28.3° corresponds to the (202) plane.

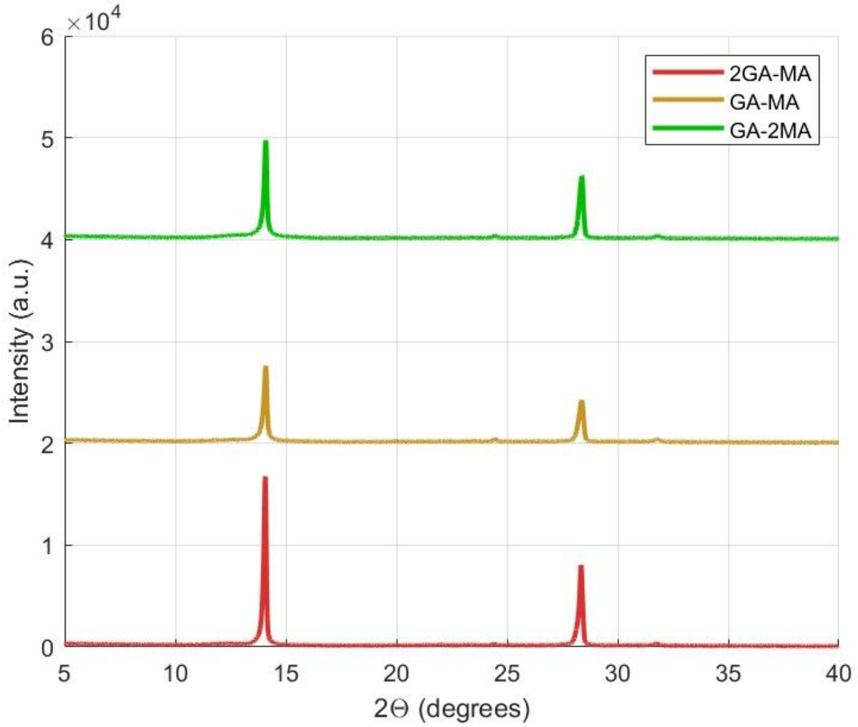

Figure S2: X-ray diffraction (XRD) patterns of the GA-based perovskite films (⟨n⟩=5).

Scanning Electron Microscopy (SEM)

Figures S3-5 show SEM images of the GA-based perovskite films (⟨n⟩=5).

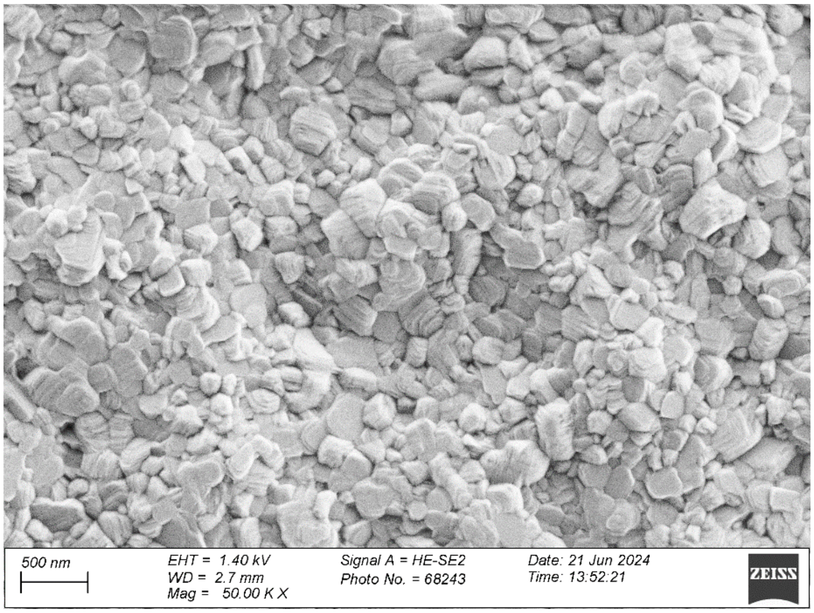

Figure S3: Scanning electron microscopy (SEM) image of the 2GA-MA perovskite film (⟨n⟩=5).

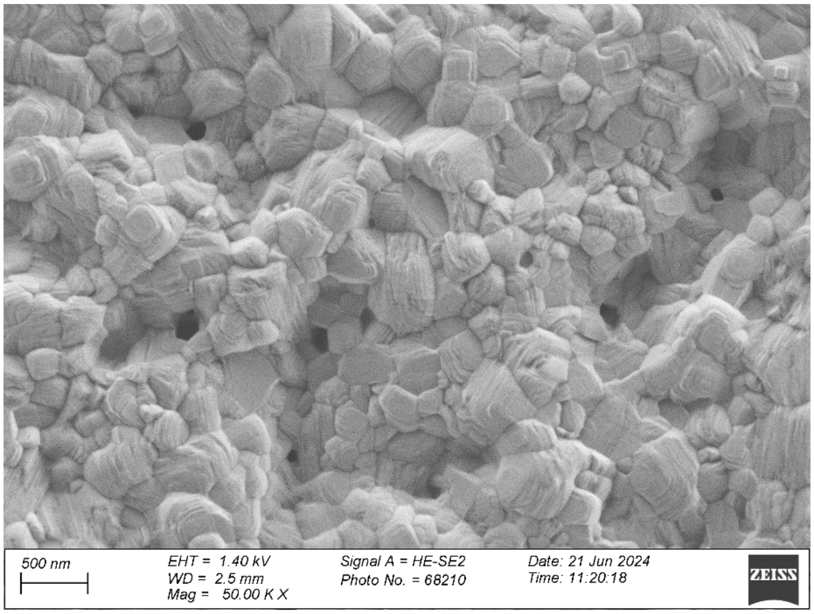

Figure S4: Scanning electron microscopy (SEM) image of the GA-MA perovskite film (⟨n⟩=5).

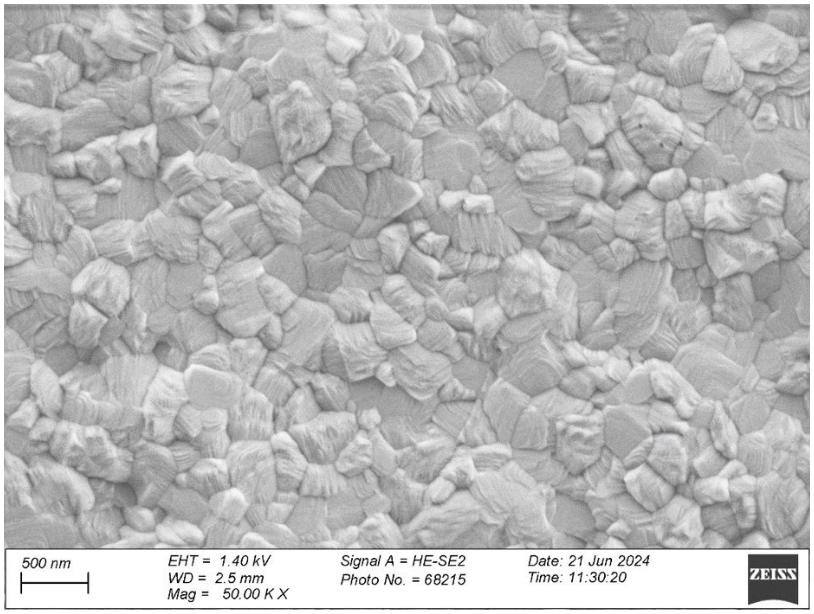

Figure S5: Scanning electron microscopy (SEM) image of the GA-2MA perovskite film (⟨n⟩=5).

TRPL Spectra Front-Side Excitation

Figure S6 shows the TRPL spectra recorded by front-side excitation. Compared to backside excitation (Figure 3 main text), the lower-n PL bands are much less prominent. This difference indicates that the lower-n domains are located nearer to the substrate side of the films.

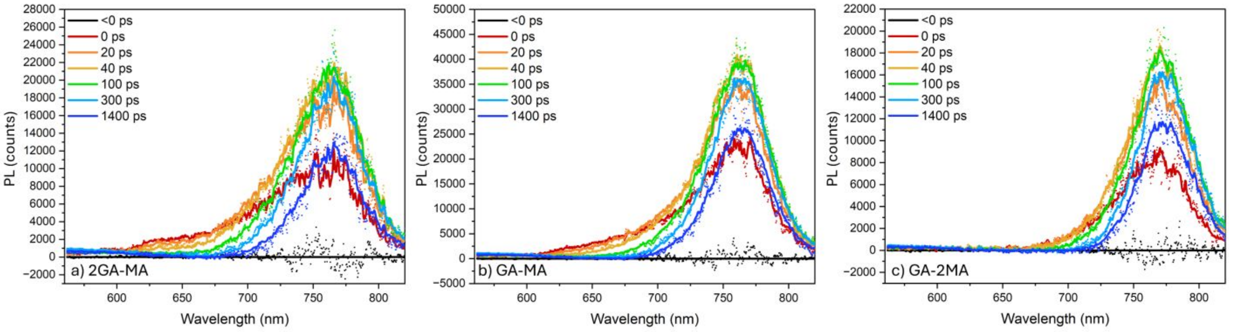

Figure S6: TRPL spectra after 532 nm front-side excitation of the a) 2GA-MA, b) GA-MA and c) GA-2MA films recorded in reflection mode. The solid lines indicate fits from photophysical modeling. The spectra are cut-off due to the use of a 570 nm long-pass filter.

TRPL Timetraces per Wavelength

Figure S7 shows the TRPL kinetic traces normalized to 1 of the films with varying GA-MA ratio at different emission wavelengths. The low-n PL (a) mainly decays within the IRT, with only a weak longer-lived component.

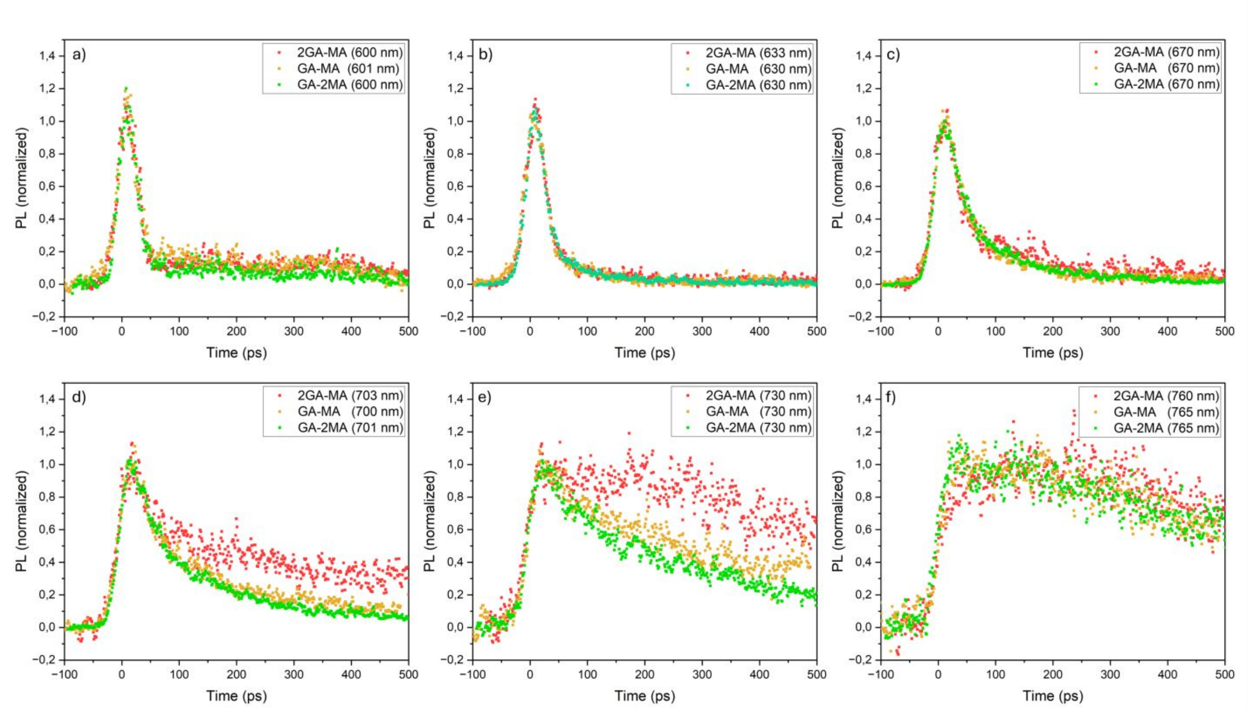

Figure S7: TRPL kinetic traces normalized to 1 of the 2GA-MA, GA-MA and GA-2MA films recorded in reflection mode around a) 600 nm, b) 630 nm, c) 670 nm, d) 700 nm, e) 730 nm and f) 760 nm.

Species Associated Spectra and Rate Constants from Target Analysis

Figures S8-S10 present the species associated spectra (SAS) obtained from the target analysis described in the main text. In the 2GA-MA and GA-MA films, the low-n SAS features two narrow bands around 600 and 630 nm, corresponding to the PL bands of the lowest-n domains, superimposed on a broader band. In the GA-2MA film, only the broader band is present. This matches with the observation that the quenched low-n <10 ps component is not present in these two films, but instead only the high-low-n. SAS3 and SAS4 contain broader bands that are centered around ~700 and ~760 nm, corresponding to the PL bands of intermediate-n and high-n domains. Tables 1 (main text) and S1-S3 present the obtained rate constants from target analysis.

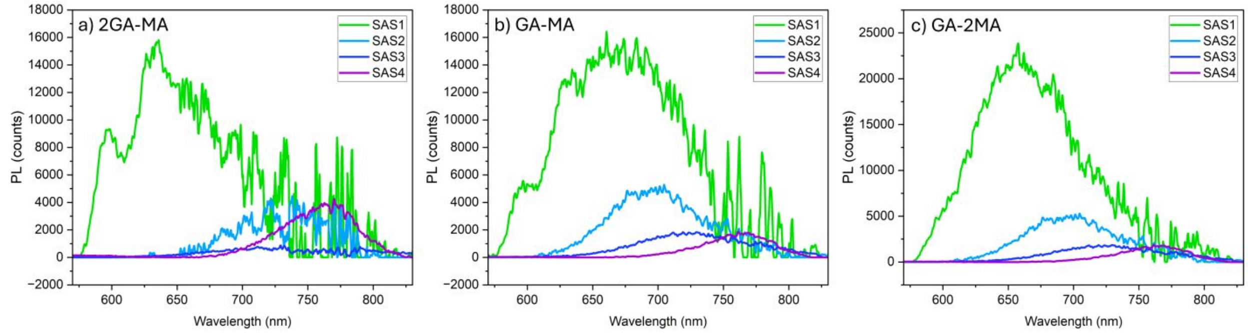

Figure S8: Species associated spectra obtained from target analysis using a 4-component sequential model on the TRPL data recorded by back-side 532 nm photoexcitation for the a) 2GA-MA, b) GA-MA, and c) GA-2MA films.

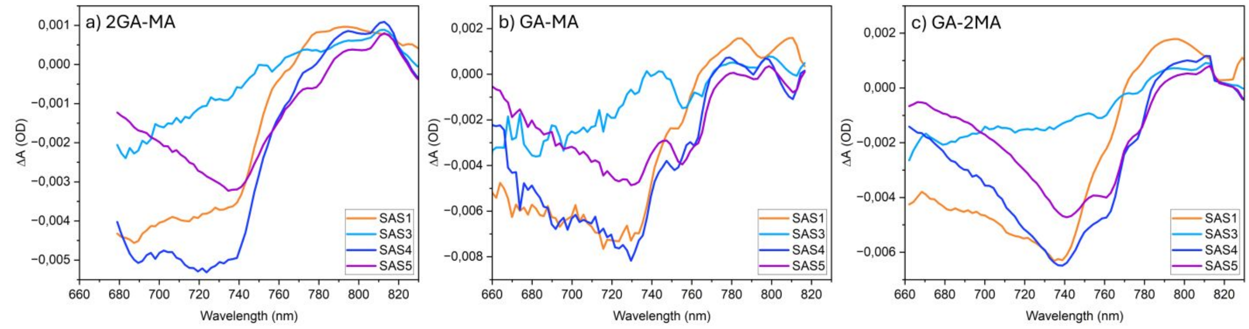

Figure S9: Species associated spectra obtained from target analysis using the photophysical model shown in Figure 9 in the main text on the TA data recorded by back-side 630 nm photoexcitation for the a) 2GA-MA, b) GA-MA and c) GA-2MA films.

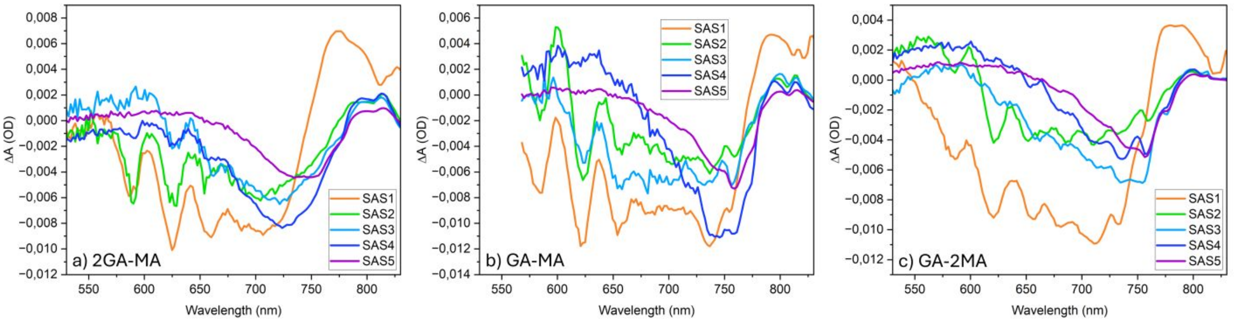

Figure S10: Species associated spectra obtained from target analysis using the photophysical model shown in Figure 9 in the main text on the TA data recorded by back-side 490 nm photoexcitation for the a) 2GA-MA, b) GA-MA and c) GA-2MA films.

Table S1: Time constants k [ps^{-1}] and τ [ps] from target analysis of the TRPL data with 3 parameters, based on the model described in the main text.

Film	k_1 [ps^{-1}]	k_2 [ps^{-1}]	k_3 [ps^{-1}]	k_4 [ps^{-1}]	k_5 [ps^{-1}]
2GA-MA	-	0.1 (f*)	-	6.3×10^{-3} (± 6.4×10^{-5})	2.39×10^{-3} (± 1.7×10^{-5})
GA-MA	-	0.1 (f*)	-	1.0×10^{-2} (± 5.8×10^{-5})	1.79×10^{-3} (± 4.9×10^{-6})
GA-2MA	-	0.1 (f*)	-	1.1×10^{-2} (± 2.7×10^{-5})	1.77×10^{-3} (± 4.1×10^{-6})
	τ_1 [ps]	τ_2 [ps]	τ_3 [ps]	τ_4 [ps]	τ_5 [ps]
2GA-MA	-	10(± -)	-	158.8(±1.6)	418.7(±3.2)
GA-MA	-	10(± -)	-	95.4(±5.1)	559.5(±2.4)
GA-2MA	-	10(± -)	-	93.6(±2.9)	565.9(±2.2)

* Decay occurs within the IRT.

Table S2: Time constants k [ps^{-1}] and τ [ps] from target analysis of the TRPL data with 4 parameters, based on the model described in the main text.

Film	k_1 [ps^{-1}]	k_2 [ps^{-1}]	k_3 [ps^{-1}]	k_4 [ps^{-1}]	k_5 [ps^{-1}]
2GA-MA	-	0.1 (f*1)	4.6×10^{-2} (f*2)	6.3×10^{-3} (f*3)	2.64×10^{-3} (±6.5×10^{-6})
GA-MA	-	0.1 (f*1)	1.9×10^{-2} (f*2)	1.0×10^{-2} (f*3)	2.07×10^{-3} (±7.2×10^{-6})
GA-2MA	-	0.1 (f*1)	2.3×10^{-2} (±2.7×10^{-4})	7.1×10^{-3} (±7.4×10^{-5})	2.06×10^{-3} (±1.2×10^{-5})
	τ_1 [ps]	τ_2 [ps]	τ_3 [ps]	τ_4 [ps]	τ_5 [ps]
2GA-MA	-	10(± -)	21.6(±0.7*2)	158.8(±1.6*3)	378.2(±1.5)
GA-MA	-	10(± -)	53.5(±5.4*2)	95.4(±5.1*3)	482.7(±2.1)
GA-2MA	-	10(± -)	43.7(±0.7)	140.0(±2.3)	486.5(±3.9)

*1 Decay occurs within the IRT. *2 Determined from tting the TA data with 630 nm excitation. *3 Determined from tting the TRPL data with 3 parameters.

Table S3: Time constants k [ps^{-1}] and τ [ps] from target analysis of the TA data at 630 nm excitation, based on the model described in the main text.

Film	k_1 [ps^{-1}]	k_2 [ps^{-1}]	k_3 [ps^{-1}]	k_4 [ps^{-1}]	k_5 [ps^{-1}]
2GA-MA	0.435 (±1.7×10^{-2})	-	4.6×10^{-2} (±1.2×10^{-3})	6.3×10^{-3} (f*2)	1.42×10^{-4} (f*3)
GA-MA	0.858 (±6.8×10^{-2})	-	1.9×10^{-2} (±1.8×10^{-3})	1.0×10^{-2} (f*2)	1.13×10^{-4} (f*3)
GA-2MA	0.758 (± 1.3×10^{-2})	-	2.3×10^{-2} (f*1)	7.1×10^{-3} (f*1)	1.08×10^{-4} (f*3)
	τ_1 [ps]	τ_2 [ps]	τ_3 [ps]	τ_4 [ps]	τ_5 [ps]
2GA-MA	766.0×10^{-3}(±3.1×10^{-2})	-	21.6(±0.7)	158.8(±1.6*2)	7.050×10^{3}(±0.38*3)
GA-MA	388.5×10^{-3}(±3.3×10^{-2})	-	53.5(±5.4)	95.4(±5.1*2)	8.813×10^{3}(±0.18*3)
GA-2MA	439.5×10^{-3}(±7.9×10^{-3})	-	43.7(±0.7*1)	140.0(±2.3*1)	9.278×10^{3}(±0.15*3)

*1 Determined from the TRPL data with 4 parameters. *2 Determined from the TRPL data with 3 parameters. *3 Determined from TCSPC.

Time-Correlated Single Photon Counting (TCSPC)

To better quantify the emissive decay of the high-n phase and exclude potential effects from the back sweep of the streak camera on lifetimes >1 ns, time-resolved PL measurements using time-correlated single photon counting (TCSPC) confocal microscope detection with a time window of ~500 ns using 485 nm pulsed excitation at 2 MHz repetition rate were performed. Also potential charge accumulation effects will be insignificant at this lower repetition rate, likewise in the femtosecond transient absorption experiments discussed below. Overview images were made by measuring the photoluminescence >633 nm, using back-side or frontside illumination (Figure S11 to S13). In the GA-MA and GA-2MA films, the emitting material is uniformly distributed across the sample, while the 2GA-MA films have regions with minimal PL.

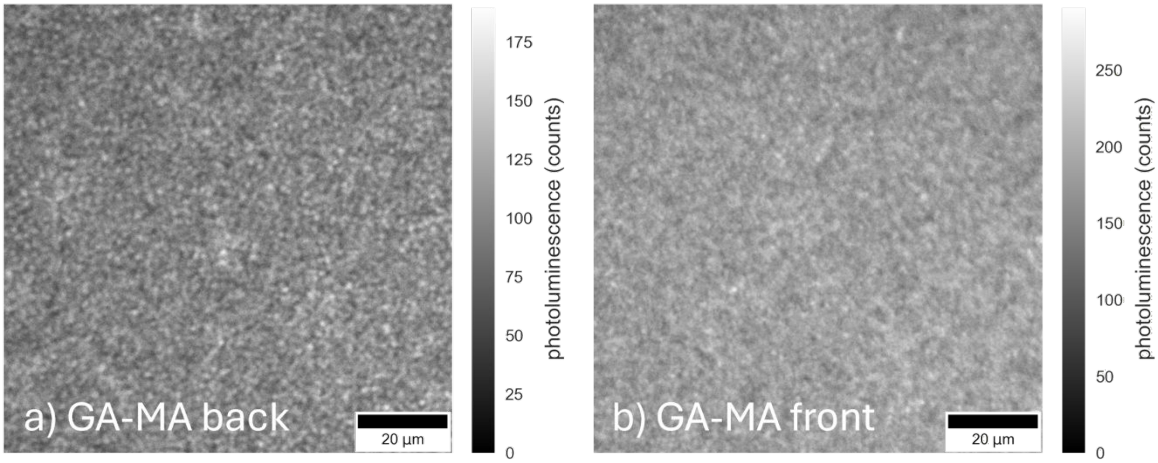

Figure S11: Confocal PL intensity maps of the GA-MA (⟨n⟩=5) film at wavelengths > 647 nm either excited from the back-side (a) or the front-side (b) of the sample recorded using 485 nm excitation.

To accurately determine the PL lifetimes, point measurements were performed at 16 (or 25) locations distributed in a 4x4 (5x5) grid over the sample area. Typical PL lifetime traces for back-side and front-side illumination at 485 nm are shown in Figures S14 and S15. The instrument response function (IRF) is reconstructed the following. After the peak of the decay, the initial and fastest part slope in the multi exponential decay is fitted to a single exponential model, from which the intensity falloff (I_{fo}) per time resolution is calculated. The IRF is then determined point by point, starting from the IRF onset, by subtracting the expected intensity at previous timepoint n-1 and the falloff from the measured intensity at timepoint n: IRF(n) = I(n) (I(n-1) - I_{fo}). This approach results in the instrument rise time and onset used for the full deconvolution t.

Average lifetimes τ_{avg} were obtained using the following formula: $\tau_{avg} = (A_1 * \tau_1^2 + A_2 * \tau_2^2 + A_3 * \tau_3^2 + A_4 * \tau_4^2)/$#counts, where A_n indicates the amplitude of the nth component, τ_n is the lifetime of the nth component, and #counts is the total number of measured photons on which the fit is based.

An overview of the fitted lifetimes is shown in Figures S17-S19. The TCSPC fitting results averaged over the 16 (or 25) datapoints are presented in Figure S16. The average lifetime t_{av} is found to be between 7 and 13.2 ns. Overall, t_{av} is shorter for back-side illumination. TCSPC fitting is available in Github, https://github.com/RobertMolenaarUT/Lifetime_fit.

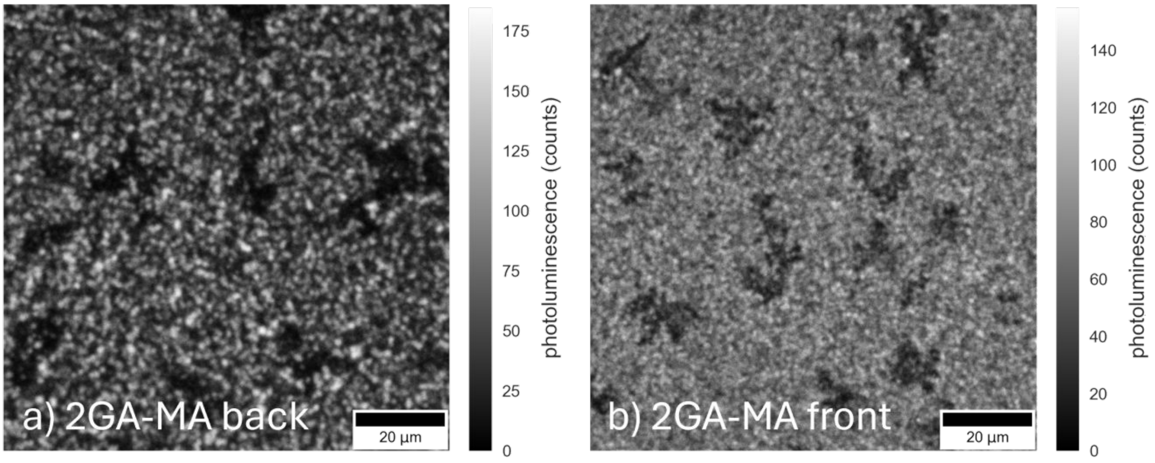

Figure S12: Confocal PL intensity maps of the 2GA-MA (⟨n⟩=5) film at wavelengths > 647 nm either excited from the back-side (a) or the front-side (b) of the sample recorded using 485 nm excitation.

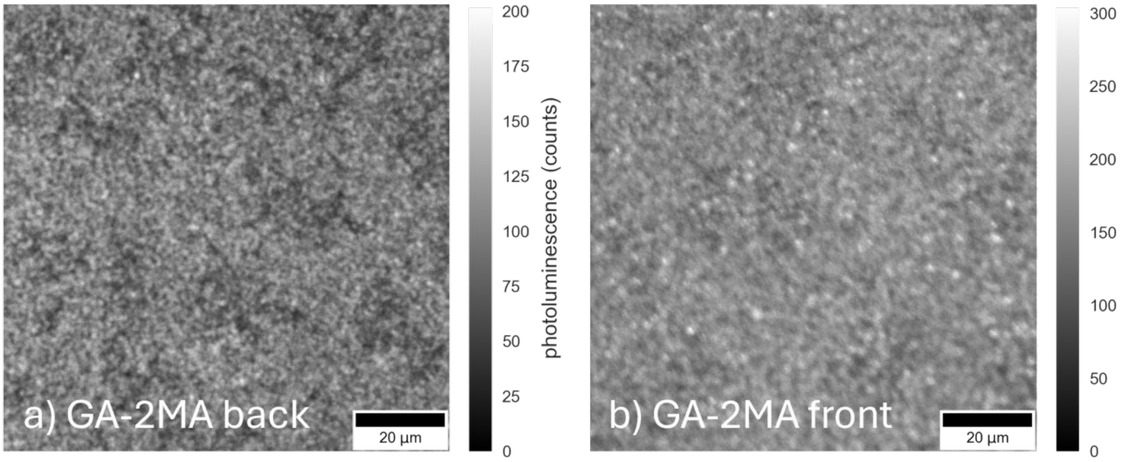

Figure S13: Confocal PL intensity maps of the GA-2MA (⟨n⟩=5) film at wavelengths > 647 nm either excited from the back-side (a) or the front-side (b) of the sample recorded using 485 nm excitation.

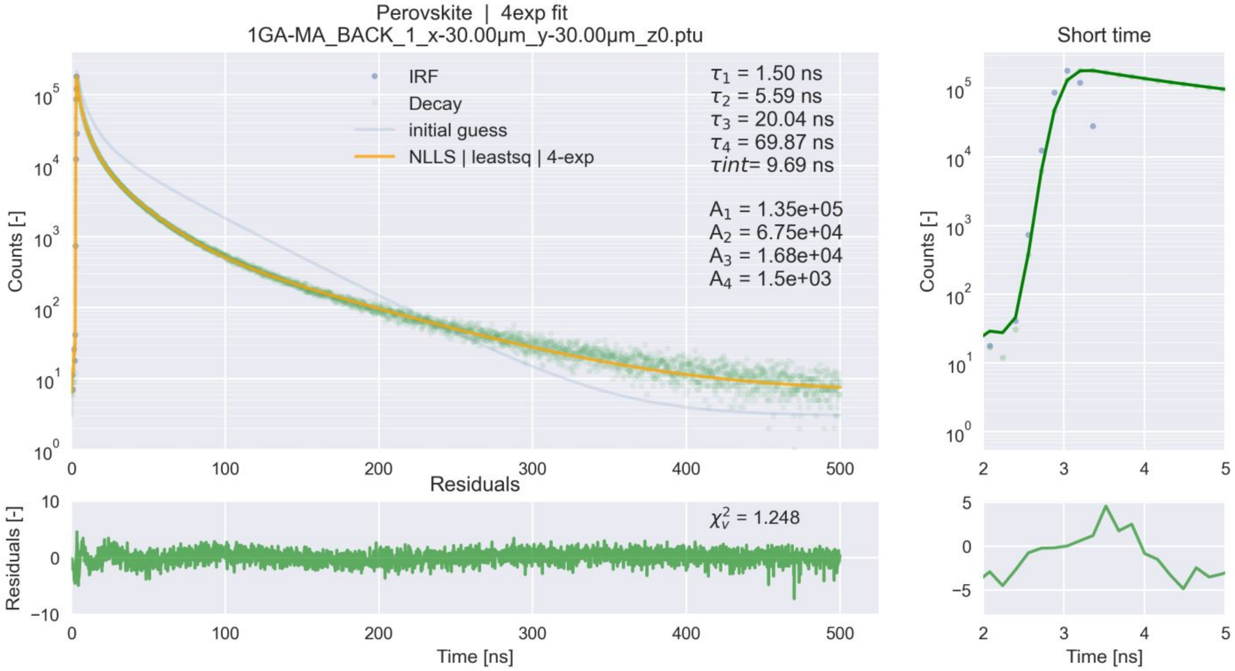

Figure S14: Typical PL decay of the GA-MA film measured by Time Correlated Single Photon Counting detection at PL wavelengths > 633 nm obtained by exciting at 485 nm from the back-side of the sample.

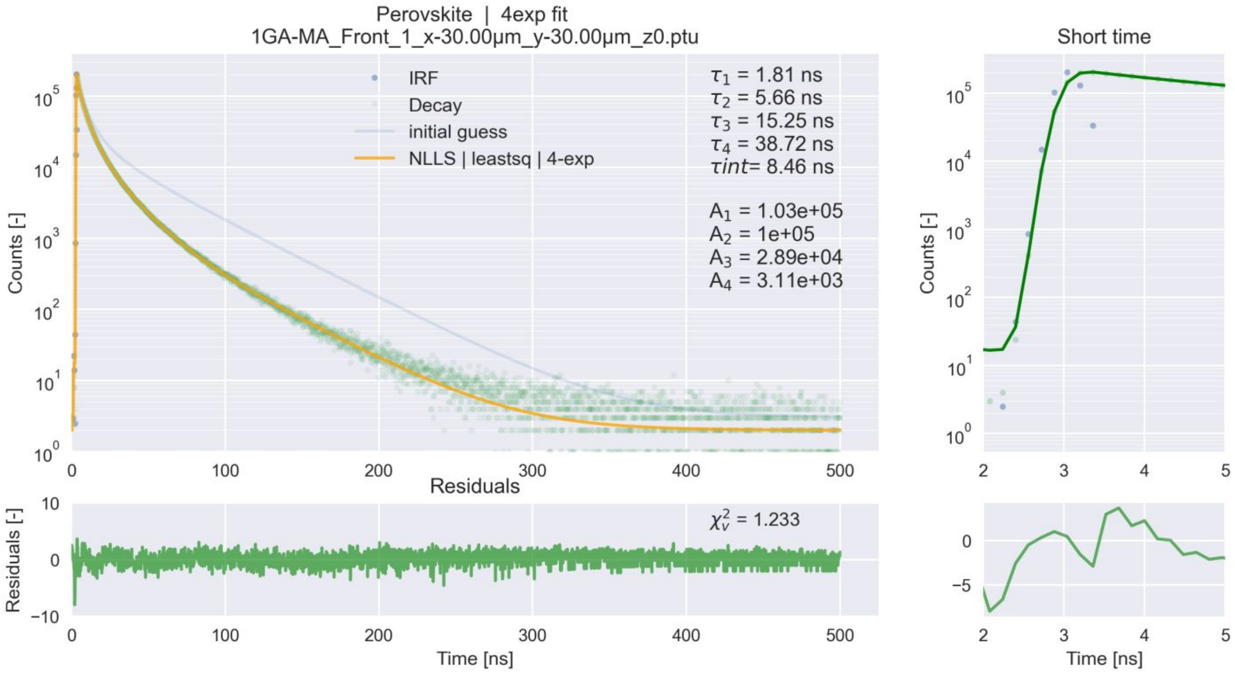

Figure S15: Typical PL decay of the GA-MA film measured by Time Correlated Single Photon Counting detection at PL wavelengths > 633 nm obtained by exciting at 485 nm from the front-side of the sample.

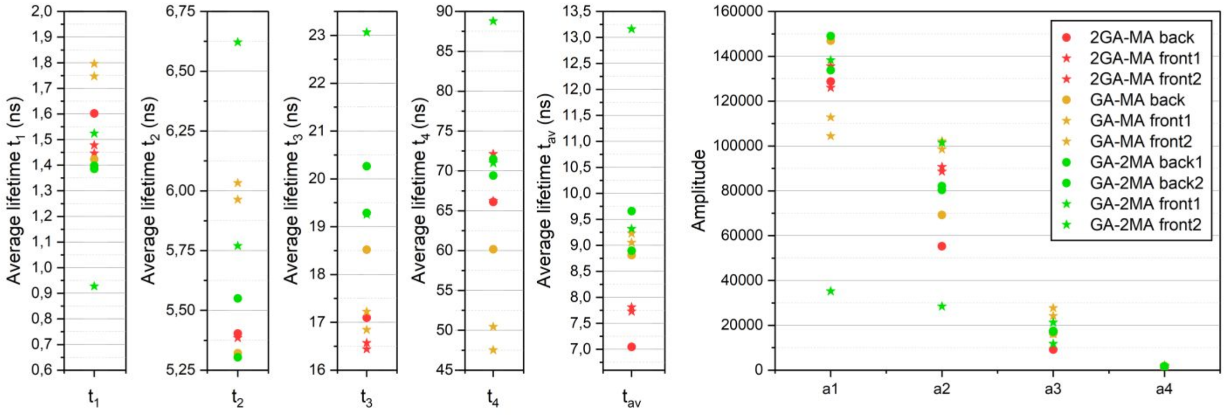

Figure S16: Average lifetimes and amplitudes from fitting the TCSPC data with the 4-exponential function described in the main text. These values were obtained by averaging over the fitting results of the respective 16 (or 25) datasets.

TCSPC Fitting Parameters

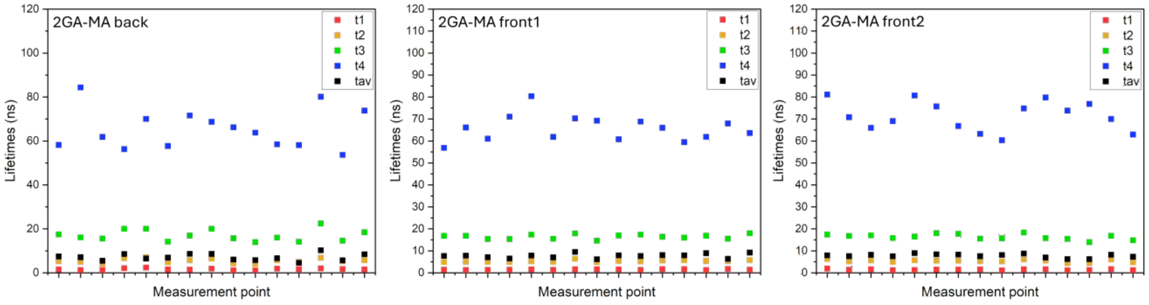

Figure S17: Obtained lifetime components after fitting the TCSPC data of the 2GA-MA film with 4 parameters under back-side and front-side excitation. t_{av} indicates the weighted average lifetimes.

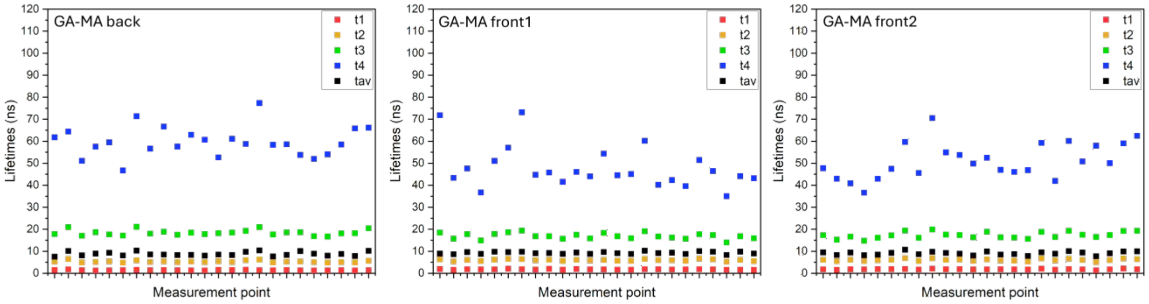

Figure S18: Obtained lifetime components after fitting the TCSPC data of the GA-MA film with 4 parameters under back-side and front-side excitation. t_{av} indicates the weighted average lifetimes.

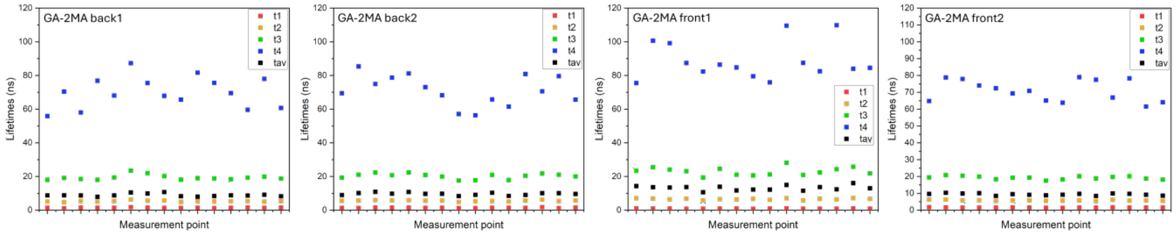

Figure S19: Obtained lifetime components after fitting the TCSPC data of the GA-2MA film with 4 parameters under back-side and front-side excitation. t_{av} indicates the weighted average lifetimes.
